# Study of Cold-Pressed Tobacco Seed Oil Properties by Gas Chromatography Method

**DOI:** 10.1155/2020/8852724

**Published:** 2020-11-10

**Authors:** Murat Z. Ashirov, Ubaidilla M. Datkhayev, Diyas A. Myrzakozha, Hidetoshi Sato, Kairat S. Zhakipbekov, Nurgali A. Rakhymbayev, Bolat N. Sadykov

**Affiliations:** ^1^Department of Organization and Management and Economics of Pharmacy and Clinical Pharmacy, School of Pharmacy, Asfendiyarov Kazakh National Medical University, Almaty, Kazakhstan; ^2^Department of Biomedical Chemistry, School of Science and Technology, Kwansei Gakuin University, Kobe, Japan

## Abstract

A special place among vegetable oils was occupied by natural tobacco oil. Natural tobacco oil in folk medicine is used as an antiseptic and antibacterial agent. To determine the possible alternative use of tobacco, the seeds representing Kazakhstan tobacco cultivars, extracted from ground *Nicotiana tabacum* seeds, were investigated by using the cold press. The quality of the oil was assessed in terms of free fatty acid content. The percentage oil yield was 36.75 ± 0.50%. Tobacco seed oil is highly unsaturated, nonacidic, and would require little purification. The oil can be used for the production of soap, antiwrinkle cream for the face, hair shampoo, shoe cream, and as a high-resolution base oil for perfumes and light industry and can be commercialized due to its high oil yield, for use as anti-inflammatory drugs.

## 1. Introduction

Tobacco was once the currency of the Almaty region and supported southern Kazakhstan's economy. As opposed to most agricultural crops, since its earliest domestication, tobacco has been bred for leaf production and characteristics. Breeding has been selected against seed characteristics because plant energy directed to seed production detracts from leaf production. Recently, health concerns over smoking and chewing tobacco have reduced the overall demand for the crop. Between 2003 and 2009, the Southern Kazakhstan Agricultural Development Commission enacted a voluntary buy out for tobacco farmers to decrease dependency on tobacco farming, improve the diversity of farm economy, and preserve the region's farmland. Farmers in southern Kazakhstan are now turning to grow alternative crops or raise livestock in place of tobacco. However, new farm products are in need of large investment in facilities, training of staff, management, and marketing. Kazakhstan has a suitable climate for tobacco, and farmers have experienced full range of facilities for farming tobacco. Therefore, the best option for farmers may be development of a profitable alternative use of tobacco.

The seeds of the tobacco plant are very small, but they come in an extremely large quantity per plant. They can be preserved for a long time if they are stored in dry conditions, are resistant to rather high humidity at ordinary temperatures, and have a strong shell. The tobacco seeds' endosperm contains thin-walled cells rich in oil. Tobacco seed oil is a final product of seed production that contains more than 30% of seed oil after extraction [1]. Tobacco seeds contain a large number of cellulose, proteins, starch, and minerals [2]. It is widely known [3–7] that tobacco seed consists of palmitic, stearic, oleic, linolenic, and linoleic acids; various amino acids; and also saturated, unsaturated, monounsaturated, and polyunsaturated fatty acids [8–12]. Hence, the tobacco seed oil is classified as a linoleic acid, which falls in the category of semidrying oil. The tobacco seed oil belongs to the semidrying oil category because it contains triglycerides of acids of linoleic series. However, due to high degree of unsaturation, the tobacco oil is susceptible to autoxidation and polymerization, which results in cross-linked and tough film upon exposure to air. There has already been research into the industrial, pharmaceutical, and food use of the tobacco plant. Tobacco oil is considered as a unique product due to squalene composition. Squalene has an immunostimulatory and anticancer activities and also contributes cholesterol metabolism normalization. Research group of La Trobe Institute for Molecular Science (Australia) at the head of Marc Hulett found out NaD1 molecules in tobacco flower that the antifungal activity has been attributed to its ability to permeabilize membranes and also inhibits tumor cell growth. At present researchers of Hexima ag-biotech company (Melbourne, Australia) are carrying out preclinical trials of NaD1 [13]. Thus, the tobacco plant has demonstrated its potential value beyond traditional uses.

The study of tobacco oil properties using gas chromatography method allows to define exactly the fatty acid composition of tobacco oil [14, 15]. Tobacco seed is a byproduct from tobacco in which yield is around 1200–1250 kg per hectare area, while in the USA, it is twice less. But it is easily possible to get up to 5.000–6.000 kg per hectare area, if selection prefers seeds, not leafs. Our field studies also grow tobacco at much higher densities than those used for leaf production, in attempt to shift the emphasis from leaf to seed development by cultural practices. Improvements through conventional breeding, genetic engineering, and low input agronomic practices are transforming tobacco into a high-yielding new oil crop. It is free of nicotine, and its oil has a low proportion of saturated fatty acids and contains health-beneficial compounds such as tocopherols and sterols.

The tobacco seed oil and flour is highly packed with antioxidant. Therefore, they can be applied in the health food industry especially in the preparation of various salad dressings, sauces, and desserts. The flour can be added into muffins, cakes, and bread as well. These foods can be used to promote healthy eating among normal consumers as well as for cancer patients since it is has a good source of cancer prevention. It is not just another oil or flour, but both provide higher value products that will benefit the end user. Additionally, today's consumers are concerned with the health benefits of a certain product. More applications of the oil and flour have to be discovered in the future.

The aim of this study is to determine and widen the fatty acid composition of tobacco oil grown in Almaty region by gas chromatography method and to find its potential uses in the preparation of food and perfumery because the use of tobacco seed oil in food and perfumes preparation has not been reported before. The present research was conducted to investigate the feasibility of alternative uses of tobacco seeds for nutraceutical or functional food ingredients. The results from this work will be used to promote the alternative use of Kazakhstan tobacco cultivars.

## 2. Materials and Methods

Tobacco oil is obtained from the cold-pressed extraction of *Nicotiana tabacum* produced by an individual entrepreneur A.N. Chebotova (Almaty, Republic of Kazakhstan) (natural tobacco oil). The oil was stored at ambient temperature under nitrogen in the dark until further testing.

Thirty percent ACS-grade H_2_O_2_ was purchased from Fisher Scientific (FairLawn, NJ, USA). *α*, *γ*, *δ*-Tocopherols were purchased from EMD chemicals Inc. (San Diego, CA, U.S.A.). Ultrapure water was prepared by an ELGA Purelab ultra Genetic polishing system with <5 ppb TOC and resistivity of 18.2 mg (Lowell, MA, U.S.A.) and was used for all experiments. All other chemicals and solvents were of the highest commercial grade and used without further purification. Sample preparation for gas chromatography was performed as follows: 0.01 g of sample was placed into test tube, and 1 сm^3^ of 2% concentrated sulfuric acid solution in butanol was added. Tightly capped test tube was placed into a drying chamber at 105°С. Butylation was carried out for 30 minutes. Then, the test tube was chilled to room temperature, and 5 сm^3^ hexane and 20 сm^3^ distilled water were added. The sample mixture was shook vigorously and left until full-phase separation. After careful removal of 1 *µ*l aliquot of the hexane layer, it was injected into the gas chromatograph. Gas chromatography was performed on Shimadzu model GC2010PLUS gas chromatograph (Kyoto, Japan), equipped with flame ionization detector (FID) and fitted with a fused silica capillary column (30 m × 0.25  mm i.d., 25 *µ*m, Supelco). Chromatograph conditions were as follows: initial column temperature 60°С programmed to increase up to 250°С. Hydrogen was used as carrier gas at a flow rate of 43,3 ml/min. Quantification was based on the area under each fatty acid peak as compared to the total area of all fatty acid peaks.

Tocopherols were determined according to a reported protocol. The tobacco seed oil was dissolved in methyl-tert-butyl ether and filtered through a 0.45 *μ*m filter. The stationary phase was Waters C-30 column (250 × 4.6 mm, 5 *μ*m). The mobile phase consisted of methanol/MTBE/water (81 : 15 : 4, v/v/v) (solvent A) and MTBE/methanol (91 : 9, v/v) (solvent B). The mobile phase was run from 0 to 16.7% solvent B in 15 min and 100% solvent B from 15 to 25 min and re-equilibrated with 100% solvent A from 25 to 30 min. The flow rate was 1 mL/min, and injection volume was 40 *μ*L. Tocopherol profile of each sample was detected at a wavelength of 295 nm and compared to known standards for quantification. All measurements were taken in triplicate.

## 3. Results and Discussion

**Figure 1:**
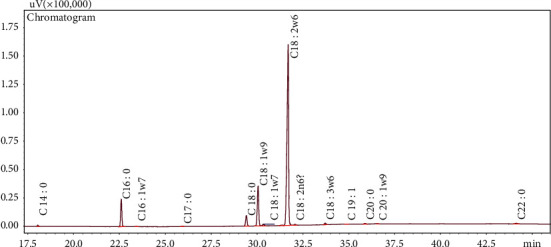
Chromatogram of fatty acid composition in tobacco seed oil.

The oil content of the seeds was found to be relatively high, i.e., 36.75 ± 0.50% on dry weight basis, using cold-press extraction, as the best method to get the tobacco seed oil, compared to other methods [8]. This high oil content of tobacco seed is comparable with that of other vegetable seeds. Tobacco seed oil appeared in yellow to light yellow color.  Figure 1 shows the peaks of the chromatogram obtained with the reference solution.

In the Table 1 the assessment of observed peaks in the chromatogram of tobacco oil is shown.

The sum of the areas of the peaks in the chromatogram, except those of the solvent, was set at 100 percent. The content of the constituent was calculated by determining the area of the corresponding peak as a percentage of the sum of the areas of all the peaks.

The amounts of unsaturated and saturated fatty acids as reported in the present work are close to those reported by Giannelos et al. and Mukhtar et al. [16, 17] (85.1% and 14.1%). However, the results of the present work differ from those of Zalatanov et al. [18] who have reported 73.9% of unsaturated and 26.1% of saturated fatty acids in tobacco seed oil. The slight difference in the amounts of different fatty acids as shown may be due to different species of tobacco (*Nicotiana*) used in the studies or due to different environmental or geographical conditions.

It is a general agreement that only acids with even number of carbons occur naturally, but it was found that tobacco seed oil contained little amounts of nonadecylic acid (0.07%) and margaric acid (0.09%) containing odd number (19 and 17, respectively) of carbon atoms. According to the results shown in Table 1, fatty acids are the most dominant in tobacco seed oil. Mono- and polyunsaturated fatty acids' domination was strongly influenced by the higher content of oleic (*ω*-9) and linoleic acids, which totally constituted 85.5%. In the case of saturated fatty acids, palmitic and stearic acids were the most dominant in tobacco oil (11.7%). Due to the high content of unsaturated fatty acids, tobacco oil has antioxidant, antiviral, immune-simulating and wound healing activities. Linoleic acid is an essential fatty acid which cannot be synthesized in human body. It is the most prevalent polyunsaturated fatty acid in all of the vegetable oils, with the highest level observed in tobacco seed oil. The linoleic acid in tobacco seed oil is about 1.5 times higher than that in soybean oil (42–53%), while olive oil has less than 5% of linoleic acid. The very high level of linoleic acid showed the potential for tobacco oil to be a potential dietary source of essential fatty acids. Oleic acid, which is the major fatty acid in olive oil (>80%), is believed to lower the incidence of cardiovascular disease [19, 20].

The highest level for *α*-tocopherol was recorded in tobacco seed oil (4.8 mg/kg oil). It also contained the highest level of *γ*-tocopherol (88.3 mg/kg oil). Tested oil also had the highest total tocopherol content (217.7 *μ*mol/kg oil). Total tocopherol is reported in *μ*mol/kg due to the different molecular weights of *α*- and *γ*-tocopherol isomers. Previous research on different Bulgarian and U.S.A. tobacco varieties showed a wide range of total tocopherol content (11.2–166.9 *μ*mol/kg) [18, 21] The tocopherol values of Kazakhstan tobacco varieties in the present work were comparable to those of Bulgarian tobacco varieties, grapeseed (37.1–263.4 *μ*mol/kg) and palm (9.3–1478.2 *μ*mol/kg) oils, but lower than those of soybean oil (234.5–6353.9 *μ*mol/kg) and some varieties of the olive oil (95–755 *μ*mol/kg). Although different in total tocopherols, the tobacco seed oils had a similar ratio between their *α*- and *γ*-tocopherols.

High levels of unsaturated fats, tocopherol, and phospholipids, a significant amount of antioxidants, and the effect on the growth of cancer cells indicate that high-yielding tobacco seed oil can be obtained with a special fatty acid composition or other useful components and serve as the basis for creating perfumes, cosmetics, and pharmaceuticals. Tobacco seed oil can serve as a source of natural antioxidants and contain antiproliferative components. The development of perfumes, cosmetics, and pharmaceuticals for health from these oils has the potential to become an excellent alternative to the use of tobacco grown in Kazakhstan. The results of this study may also be applicable to tobacco cultivation in other countries. Additional research is needed to further investigate the effects of tobacco seed oil formulation, processing, and storage on the availability of beneficial components and properties, as well as phytochemical components and biochemical mechanisms involved in antiproliferative properties, in order to better use the oil for health promotion and disease prevention.

The high level of unsaturated fat, tocopherol, and phospholipid, significant amount of antioxidants, and effect on growth inhibition of cancer cells indicated that the high-yield tobacco seed oil may be obtained with special fatty acid composition or other beneficial components and serve as edible oil. The tobacco seed flours may provide dietary sources of natural antioxidants and contain antiproliferative components. Development of health products from these flours and oils has the potential to be excellent alternative uses of Kazakhstan grown tobacco. The results of this research may also be applicable to tobaccos growing in other countries. Additional research is still required to further investigate the effects of food formulation, processing, and storage on the availability of these beneficial components and properties, as well as the phytochemical components and biochemical mechanisms involved in antiproliferative properties, in order to better utilize the oil and flour for health promotion and disease prevention.

## 4. Conclusions

From the present study, it is concluded as follows:

1. The tobacco seeds were rich in oil content containing linoleic acid (71.73%) and oleic acid (13.77%) as the most abundant fatty acids.
2. The anticancer properties and aroma holding capacity of tobacco seed oil attributed to particular unsaturated fatty acid composition of tobacco seed oil [21]. The properties such as high unsaturated fatty acid content and low saturated fatty acid content of tobacco seed oil make it particularly suitable for the manufacture of new line of perfumes, and industrial salad dressings and can be used as an emulsifier in food processing and high penetration massage oil. Tobacco seed oil could be used as a solvent for its own flowers and selected for its scent. Its scent is sweet without being cloying, evocative of old-fashioned cottage gardens.

In order to examine the fatty acid composition in tobacco oil, an experiment was carried out by gas chromatography. The experiment showed that the higher content of unsaturated fatty acids was observed compared to saturated fatty acids, 85.5% and 11.7%, respectively.

## Figures and Tables

**Table 1:** Fatty acid composition in tobacco seed oil.

№	Fatty acid	Quantitative content (%)
1	Myristic	0.2853
2	Palmitic	8.0426
3	Palmitoleic	0.0943
4	Margaric	0.0934
5	Stearic	3.6680
6	Oleic (*ω*-9)	13.7773
7	Vaccenic	0.6733
8	Linoleic	71.7331
9	Linoelaidic	0.3489
10	**α**-Linolenic	0.5341
11	Nonadecylic	0.0701
12	Arachidic	0.2298
13	Gondoic	0.1134
14	Behenic	0.3364

## Data Availability

The data used to support the findings of this study are included within the article.
